# Population health management in France: specifying population groups through the DRG system

**DOI:** 10.1186/s12913-021-06757-x

**Published:** 2021-07-24

**Authors:** A. Malone, S. Gomez, S. Finkel, D. Chourchoulis, E. Morcos, M. A. Loko, T. Gaches, D. Laplanche, S. Sanchez

**Affiliations:** 1 French Hospital Federation, Paris, France; 2 Departement d’Information Médicale, CHU Clermont-Ferrand, Clermont-Ferrand, France; 3 Département d’Information Médicale, Centre Hospitalier de Douai, Douai, France; 4 Département d’Information Médicale, Centre Hospitalier de Vesoul, Vesoul, France; 5 Département d’Information Médicale, Centre Hospitalier de Dax, Dax, France; 6 Département d’Information Médicale, Centre Hospitalier de Chartres, Chartres, France; 7 Pôle Territorial Santé Publique et Performance, Hopitaux Champagne Sud, Troyes, France; 8 Universitary comity of ressources for research in health (CURRS) University of Reims Champagne-Ardenne, 51 Rue Cognacq Jay, 51095 Reims Cedex, France

**Keywords:** DRG, Accountable care organization, Population health management, Territorial hospital group

## Abstract

**Background:**

Population health management (PHM) by hospital groups is not yet defined nor implemented in France. However, in 2019, the French Hospitals Federation launched a pilot program to experiment PHM in five territories around five Territorial Hospital Groups (GHT’s). In order to implement PHM, it is necessary to firstly define the population which healthcare facilities (hospitals) have responsibility for. In the French healthcare system, mapping of health territories however relies mainly on administrative data criteria which do not fit with the actual implementation of GHT’s. Mapping for the creation of territorial hospital groups (GHTs) also did not include medical criteria nor all healthcare offers particularly in private hospitals and primary care services, who are not legally part of GHT’s but are major healthcare providers. The objective of this study was to define the French population groups for PHM per hospital group.

**Methods:**

A database study based on DRG (acute care, post-acute and rehabilitation, psychiatry and home care) from the French National Hospitals Database was conducted. Data included all hospital stays from 1 January 2016 to 31 December 2017. The main outcome of this study was to create mutually exclusive territories that would reflect an accurate national healthcare service consumption. A six-step method was implemented using automated analysis reviewed manually by national experts.

**Results:**

In total, 2840 healthcare facilities, 5571 geographical zones and 31,441,506 hospital stays were identified and collated from the database. In total, 132 GHTs were included and there were 72 zones (1.3%) allocated to a different GHTs. Furthermore, 200 zones were manually reviewed with 33 zones allocated to another GHT. Only one area did not have a population superior to 50,000 inhabitants. Three were shown to have a population superior to 2 million.

**Conclusions:**

Our study demonstrated a feasible methodology to define the French population under the responsibility of 132 hospital groups validated by a national group of experts.

## Background

Better health, better care and at the best cost for a given population, known as the Triple Aim, is a key organizing principle for many health systems and organization [1, 2]. As such, population health management (PHM) has become a key operational tool contributing to the Triple Aim [3].

Both the Triple Aim and PHM require three key components from a healthcare provider point of view. The first is a shift from a reactive, individual patient-based approach towards a pro-activepopulation-based strategy. In practice, this would be through providers being accountable not only for the care of individual patients that may show up at any given location but also attending to a larger population with the objective of keeping it as healthy as possible. The second component would be the need for an “integrator”, which may be an organization leading, launching and supporting this shift towards the new model of care for a given population [4]. This in turn would lead to the third key component, which is the ability to define which population a given set of providers would be held accountable for.

Far from being straightforward, the question of accountability has received at least two types of answers. Accountability may be defined through affiliation or geographical basis. In the Accountable Care Organization (ACO) models developed in the US, providers are accountable for the well-being of a population determined through its affiliation with a given health insurance scheme [5, 6]. In the UK, on the other hand, the development of the PHM is through a geographical basis. Integrated care systems currently being rolled out in England (NHS, 2021) hold providers accountable for the well-being of all inhabitants of a given area [7].

Population-based health policies have gained traction in France in recent years, although neither the Triple Aim nor PHM are explicitly endorsed be national authorities [8]. Even though France has universal health coverage, as well as regional health planning authorities (*Agences Regionales de santé),* providers still work largely on the “individual patient” basis where each provider cares for a specific health need of a specific patient with minimal done in terms of coordination and integration [9, 10].

The French Hospitals Federation developed a PHM model based on the concept of a shared population accountability (*Responsabilité populationnelle*), in which the newly formed Territorial Hospital Groups (GHTs) would play the role of integrators [11, 12]. Starting since 2018, it has launched a large-scale study aiming to develop and test the model in five GHTs, representing a population of around 1.5 million people.

Territorial hospital groups include all public acute-care hospitals in a given territory, as well as some long-term retirement homes, public mental health providers, and associated services. However, from a PHM perspective, GHTs suffer from at least three drawbacks. The first is that contrary to what the name implies, they do not strictly have defined geographical boundaries. From a legal standpoint, GHTs are formed through a list of “providers” (i.e. GHT for region X is formed of hospitals A, B and C), which may or may not cut across administrative boundaries such as “*departements”*. Patients are free to seek care with any provider, including private facilities that are not part of the GHT, and public hospitals belonging to another GHT*.* There are 132 GHTs covering the 100 *departements* in France. It is therefore unclear where accountability for a population starts and where it ends. The second drawback is that GHTs include only public providers, whereas private sector hospitals and ambulatory providers represent a large activity in France. Lastly, patients have the legal freedom to seek care wherever they choose. Focusing only on patients that visit a given care provider may lead to a distorted picture of the true healthcare needs in a given place. However, in spite of these drawbacks, GHTs remain the preferred building block for a PHM approach as many providers are grouped into a single organization that is able to provide a wide range of hospital services, from preventive services to highly specialized care is a major argument. Also, private facilities and ambulatory providers tend to be located around public hospitals. Therefore creating healthcare territories around GHTs may be the most logical solution to develop a PHM model. However, as GHTs do not have, strictly speaking, geographical boundaries, it remains a key challenge to define for which population a given provider GHT is accountable. In the absence of such boundaries, the question of which GHT is accountable for which population remains open.

In order to implement a PHM model, the first priority was to develop a method by which healthcare territories would be created so that all providers would be held accountable of the well-being of the inhabitants. These territories would be built around the GHTs to be the main building blocks of the French healthcare organization, integrating private sector providers and have meaning for ambulatory providers (in particular for GPs). The objective of this study was to define the French population groups for PHM per hospital group.

## Methods

We aimed to define territories by utilizing healthcare services consumption, attributing a given area to a territory if the providers of that territory provided the majority of healthcare services. We did not focus on a single disease or a specific type of population but chose to capture total healthcare consumption from private and public hospitals. Ambulatory care providers who are located inside these areas would be also considered accountable for the population, even though it was technically challenging to link ambulatory and hospital’s services consumption. However, since ambulatory care providers cover a much smaller footprint that hospital providers and since ambulatory and hospital providers would be linked through a shared PHM approach, we did not consider this as an issue.

A retrospective study of healthcare services utilization was conducted based on Diagnosis-related Groups (DRG, acute care, post-acute and rehabilitation, psychiatry and home care), through the national PMSI (*programme de médicalisation des systèmes d’information*) database. This database records all hospitals stays and provides detailed, if anonymized, information on the patients, including their area of residence through a geographical code (Code GEO PMSI). Therefore, hospital utilization is linked with an area of residence. The database contains utilization data for all types of hospitals (public, private for profit, private non for profit) and all types of hospitalization (short stay, rehabilitation, psychiatry and hospital at home) [13].

The population attribution to the GHTs was created in a prospective approach where the attribution of a population for the coming years was based on the patient service use from previous years [14].

All hospital stays recorded in the database between 1 January 2016 and 31 December 2017 were included in the study. Hospital stays were anonymous and a single patient could have different hospital stays within the period of the study. Hospital stays were included in the study if a corresponding geographical code and at least one diagnosis code was available. The geographical codes used in the database are linked to “*communes*” (counties), the smallest administrative area in France. There are 5571 “Geo PMSI” geographical codes, and 132 GHTs. There are 34,968 “*communes*” in France, therefore a Geo PMSI code may include multiple communes. Grouping of communes inside a single Geo PMSI code happens only in the case of communes with less than 1000 residents. The main study outcome was to create mutually exclusive territories that would reflect accurate healthcare service consumption regardless of the provider type or the type within which the services were provided. In addition to the GHTs, supra-GHTs were defined to include as well private hospital services not included in the scope of a GHT, however located within the same territory. These supra-GHT are the aera where our PHM approach would be deployed. The residents of these area are the residents towards whom healthcare providers are accountable whether they utilize healthcare services or not.

Statistical analysis was conducted in a stepwise approach comprising of three consecutives steps. The first step allocated all hospital stays of resident patients within a geographical code to the GHT which provided the majority of stays for the residents of the geographical code. The allocation was made based on two methods: firstly, the number of absolute hospital stays (*séjour*) in acute hospitals and secondly the number of hospital days adjusted to the mean daily cost of a full hospital day per type of activity (short stay, rehabilitation, psychiatry and hospital at home). All daily costs were extracted from the national database. This allowed for homogenization of units of consumption between activities since rehabilitation, psychiatric and hospitals at home are not payed on the same basis and do not use stays as a unit of measure. In this step, we focused our analyses only on services provided by the GHT, excluding private providers. The formula for the first GHT in the zone in terms of number of days according to the “Total days” indicator was = C1 x nr d MCO + C2 x nr d SSR + C3 x nr d HAD + C4 x nr d Psy (the values of C are the average costs: 547 for medicine, surgery and obstetric, 220 rehabilitation, 140 home hospitalisation and 310 for psychiatry)This allowed us to identify a leading GHT in each geographical code. In a small number of areas where the identification of a leading GHT was difficult, such as in borders areas between two GHTs, expert opinion was used. Each geographical code where a GHT was the leader by more than 10% in terms of services consumption compared to a neighbouring GHT was automatically attributed to the leading GHT. Below that threshold, decision was taken based on expert advice. Decision was also taken to include in a GHT territory all areas within a territory.^1^

At the end of this first step, each geographical code was attributed to a single GHT. Grouping several codes defined a territory, which could be linked to “*communes*” (counties). Therefore, in this first step, the territory of a GHT is formed by all the counties where hospitals that belong to that GHT are the leading providers.

The second step involved adding all the other hospital providers (private for profit, private non-profit) inside the geographical codes attributed to a GHT, to assess if it changed the pattern of consumption and therefore the leading GHT in a given geographical zone. This stage makes it possible to adjust the boundaries of the GHT territories while taking into account the private healthcare activity. The same method of homogenization of consumption between types of activities was used. A second round of expert advice was then used for consistency of results and consistency of territories.

According to French legislation, this study did not require patient consent. An authorization from the French Data Protection Authority (CNIL) was obtained (no.715016) to have access to the French national hospital database.

## Results

**Fig. 1:**
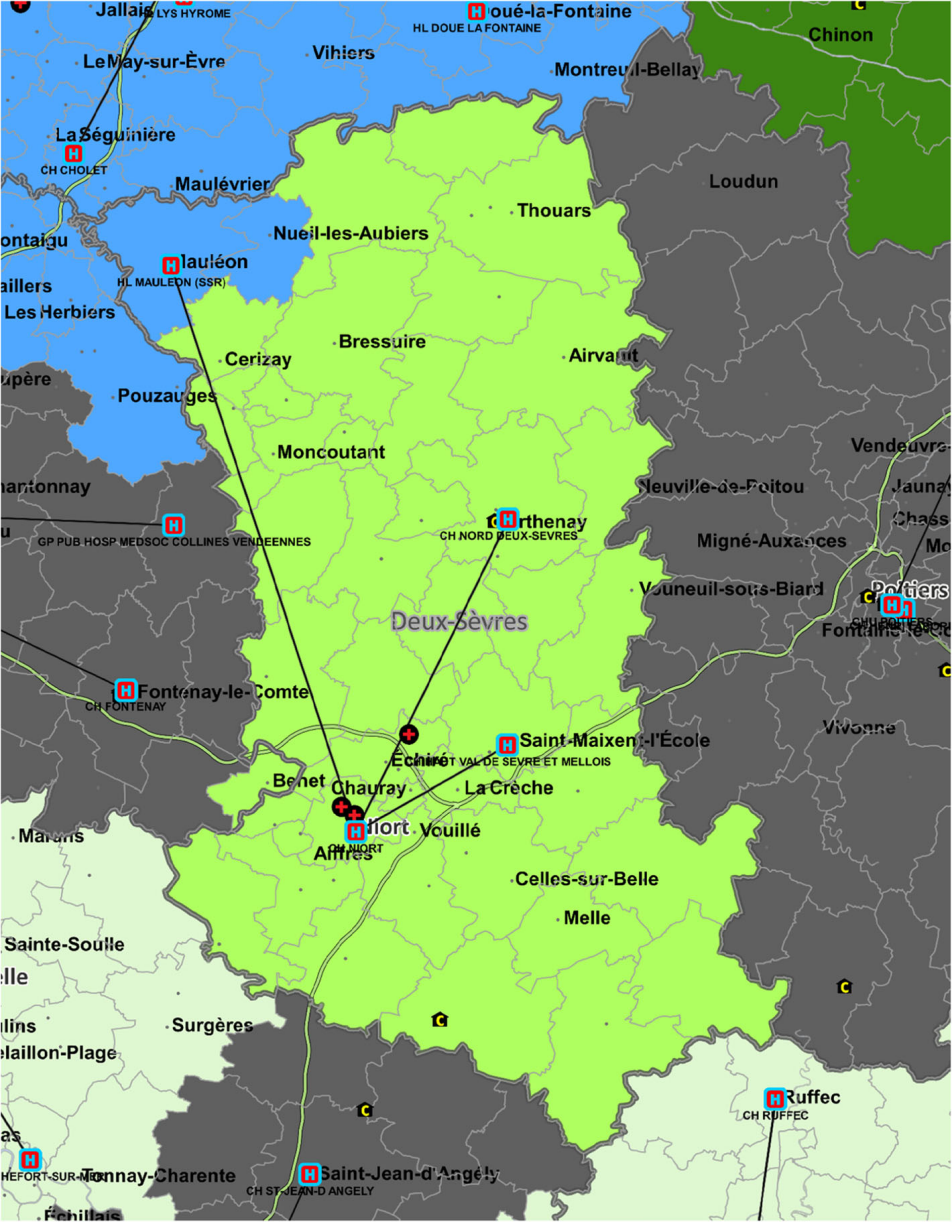
Geographical zone allocation case example for the territorial hospital group (GHT) in the French department of Deux-Sèvres. An area around a facility belonging to a GHT (shown by the lines) was attributed to another GHT since healthcare utilization was overwhelmingly directed towards the latter

In total, 2840 healthcare facilities, 5571 geographical zones and 31,441,506 hospital stays were identified in the database. In the first step, 132 GHTs were included corresponding to 14,992,531 stays. The rest of the stays were performed in private hospitals. There were 72 zones (1.3%) allocated to a different GHT according to the two allocation methods. In the second step, 200 zones were manually reviewed by the group of experts including 72 zones that were previously identified. Among the 200 zones, 33 zones were allocated to another GHT than the one defined in the first step. The example of the GHT in the French department Deux-Sèvres is shown in Fig. 1 and it is worth noting that the zone for the facility in Mauleon, which legally belongs to the Deux-Sèvres GHT was attributed to the neighbouring GHT since the overwhelming majority of healthcare utilization of the residents of that area took place in the second GHT. This decision was made after expert review. Mauleon hospital is a small long term care facility with limited medical services. Therefore, for usual healthcare needs, the residents of that area go to the closest general hospital, which belongs to another GHT.

**Fig. 2:**
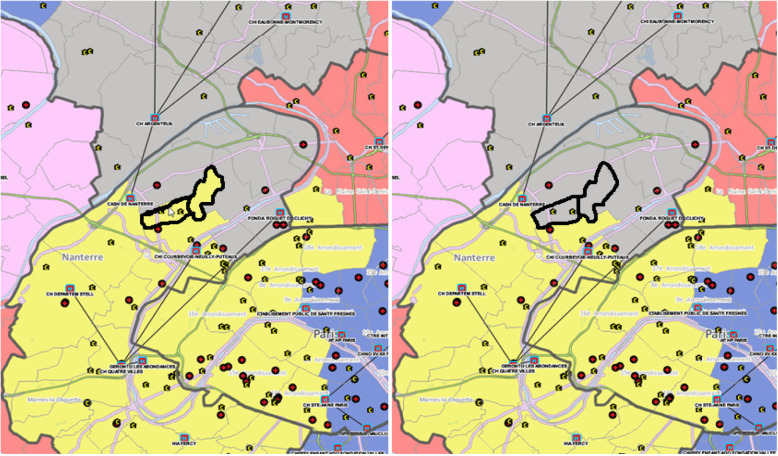
Change in geographical zone attribution. Before and after images of the territorial hospital groups (GHT) in the Hauts-de-Seine department of France. Expert review led to a change in attribution

Regarding the second step, which consisted of integrating non-public hospitals in the supra-GHT, 29 zones were identified as having differences equal or inferior to 10% between the first and second allocated supra-GHTs. Among the 29 zones, six were randomly chosen to be thoroughly reviewed by the experts. Among the six reviewed zones, five were allocated to the same supra-GHTs as recommended by the automated analysis and one zone was attributed to a different supra-GHT than the one recommended by automated analysis. This change was made to the department of Hauts-de-Seine near Paris where the geographical zone of Bois-Colombes was allocated to another supra-GHT area after the expert review (Fig. 2). This choice was made to avoid a “donut hole” effect.

**Fig. 3:**
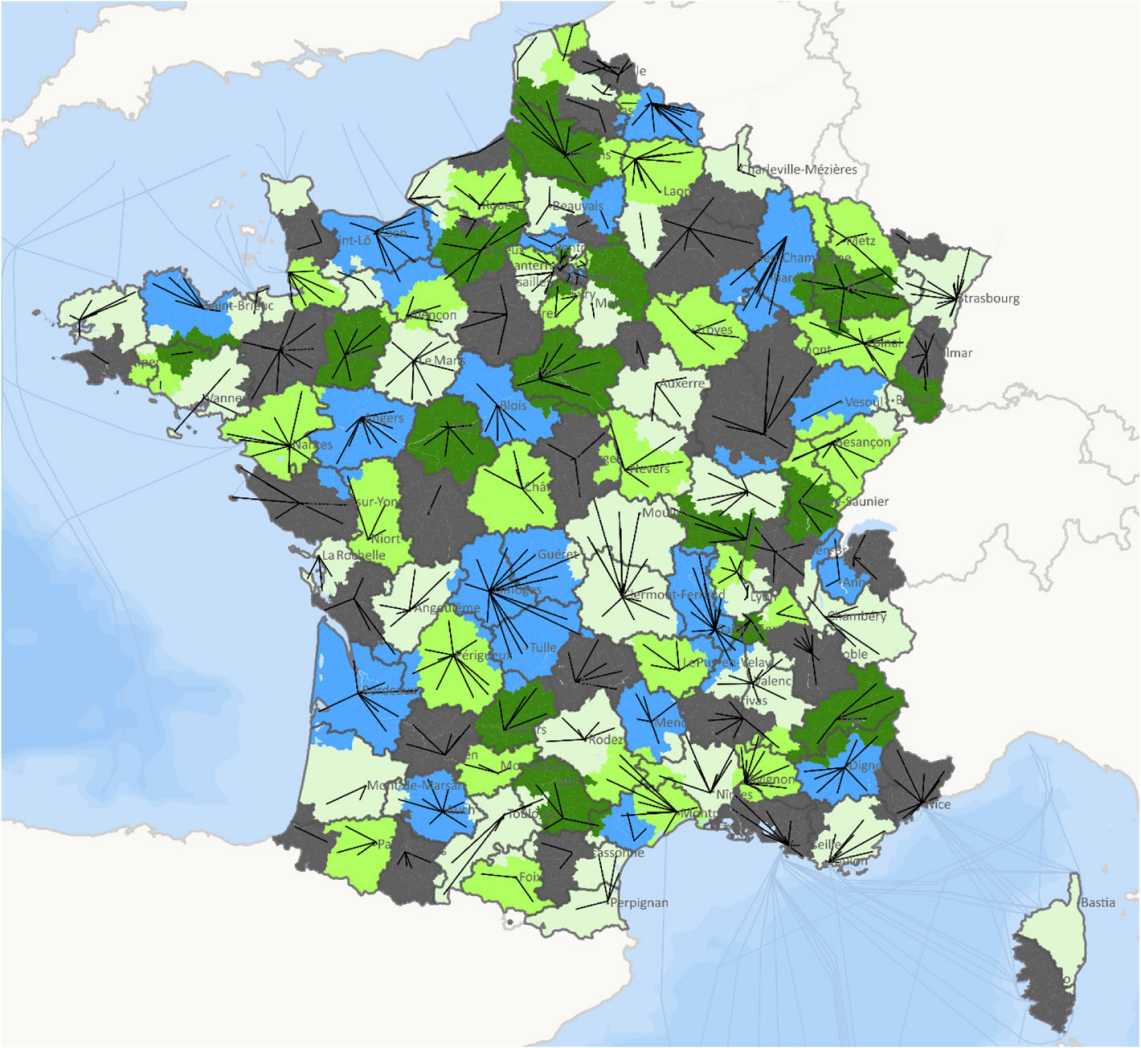
National map of the territorial hospital groups (GHTs) in France. Source: PMSI 2017 Atih – FHF (French Hospital Federation) Data

In terms of population, 131 supra-GHT areas over 132 had an attributed population superior to 50,000 inhabitants. The area with a population less than 50,000 was in Saint-Martin which is an overseas French territory located in the Caribbean with a population less than 40,000. Three areas had an attributed population more than 2 million. These three areas were in the departments: Hauts-de-Seine, 94-Nord and Bouches-du-Rhône (Fig. 3).

## Discussion

In this study, the attribution of a population under the responsibility of 132 GHTs in France was achieved using a relatively easy to use methodology and was validated by a national group of experts.

The main strength of our study relied on the use of the French national hospital database including all hospitals and all stays in France. Since we chose to rely on stays and days and homogenized the results across all types of hospitalization and not on diagnostic and technical interventions, we minimized the risk of bias associated with incomplete or wrong coding and potential errors could not have affected the results obtained [15–17]. The exploratory stepwise statistical method used allowed us to limit errors when allocating the zones to a GHT. Remaining discrepancies and unexpected results were manually checked by a group of experts in medical coding and public health. In terms of limitations, the methodology was time-consuming and should be improved to continuously update the geographical zones and GHT areas to consider the possible integration or deletion of some healthcare facilities (hospitals) within the bounds of the current mapping of GHTs. Additionally, healthcare offers and organizations could evolve within a territory due to national healthcare reforms such as the introduction of telemedicine, value-based funding and primary care communities or the opening or closing of activities within public or private hospitals [8, 18–20].

The population basis attributed to the hospital group may as well differed from year to year due to patient healthcare consumption behaviours which may potentially have induced population loss or a ‘beneficiary churn’ within the population [21, 22]. Therefore, the attribution method should also include an approach to monitor patient panel stability and the use of services within the attributed population over time, even though the population was stable with time in additional analysis [23, 24]. A classification of the hospital groups may additionally be useful in defining the areas based on the results obtained in this study. Our findings may better identify characteristics and differences between the groups and their attributed population in order to define accurate and context-driven population health intervention in the future [25].

Within the framework of the FHF initiative, which seeks to deploy a PHM approach in five GHTs, the geographical areas defined by this study will be used to extract sub-populations such as residents at risk or suffering from type 2 diabetes and chronic heart failure, which are the operational backbone of the experiment. Our study allowed us to have a fairly precise view of how many people would have specific healthcare needs and plan services in each territory including healthy but at-risk individuals.

The findings in this study are the first to allow healthcare professionals including ambulatory providers in France to have a more precise view of the population for which they are accountable. Being able to draw relevant boundaries around Supra GHTs, and using GEO PMSI codes to do so allows healthcare professionals and healthcare authorities to know precisely which counties belong to their area of accountability.

In conclusion, the geographical areas defined were used to generate relevant data for healthcare professionals in the five GHTs. These areas where well accepted by the stakeholders from those GHTs, recognized to be meaningful in their clinical work and constitute a first step in creating a shared awareness of a shared accountability. Furthermore, beyond the initiative in the five pilot territories, the methodology used in this study allowed us to have a clear picture of the precise areas covered by the 132 GHTs in France allowing FHF and public hospitals to plan for a scaling up of their PHM model to other GHTs or towards other sub-populations.

## Data Availability

Data and material can be available upon request to the corresponding author. The datasets used and analyzed are nationwide hospital in patient stay database and are also available from demande_base@atih.sante.fr (https://www.atih.sante.fr/bases-de-donnees/commande-de-bases) in respect with French legislation about access and research on nationwide database.

## Footnotes

1 For instance, if GHT “2” was leader in a zone within the territory of GHT “1”, to avoid a “donut hole” effect.
