# Correction to: Population health management in France: specifying population groups through the DRG system

**DOI:** 10.1186/s12913-021-06830-5

**Published:** 2021-08-09

**Authors:** A. Malone, S. Gomez, S. Finkel, D. Chourchoulis, E. Morcos, M. A. Loko, T. Gaches, D. Laplanche, S. Sanchez

**Affiliations:** 1 French Hospital Federation, Paris, France; 2 Departement d’Information Médicale, CHU Clermont-Ferrand, Clermont-Ferrand, France; 3 Département d’Information Médicale, Centre Hospitalier de Douai, Douai, France; 4 Département d’Information Médicale, Centre Hospitalier de Vesoul, Vesoul, France; 5 Département d’Information Médicale, Centre Hospitalier de Dax, Dax, France; 6 Département d’Information Médicale, Centre Hospitalier de Chartres, Chartres, France; 7 Pôle Territorial Santé Publique et Performance, Hopitaux Champagne Sud, Troyes, France; 8 Universitary comity of ressources for research in health (CURRS) University of Reims Champagne-Ardenne, 51 Rue Cognacq Jay, 51095 Reims Cedex, France

**Correction to: BMC Health Serv Res 21, 733 (2021)**

**https://doi.org/10.1186/s12913-021-06757-x**

Following publication of the original article [1], the affiliation numbers for the author D. Laplanche were incorrect, and should be changed from D. Laplanche^7,8^ to D. Laplanche^7^.

The author affiliation list has been updated above and the original article [1] has been corrected.

## References

[1] Malone (2021). Population health management in France: specifying population groups through the DRG system. BMC Health Serv Res.

